# MicroRNA-21 in urologic cancers: from molecular mechanisms to clinical implications

**DOI:** 10.3389/fcell.2024.1437951

**Published:** 2024-07-23

**Authors:** Lifeng Gan, Liying Zheng, Junrong Zou, Peiyue Luo, Tao Chen, Jun Zou, Wei Li, Qi Chen, Le Cheng, Fangtao Zhang, Biao Qian

**Affiliations:** ^1^ The First Clinical College, Gannan Medical University, Ganzhou, Jiangxi, China; ^2^ Department of Urology, The First Affiliated Hospital of Gannan Medical University, Ganzhou, Jiangxi, China; ^3^ Key Laboratory of Urology and Andrology of Ganzhou, Ganzhou, Jiangxi, China; ^4^ Department of Graduate, The First Affiliated Hospital of Gannan Medical University, Ganzhou, Jiangxi, China

**Keywords:** miR-21, cancer, prostate, bladder, kidney, urinary system

## Abstract

The three most common kinds of urologic malignancies are prostate, bladder, and kidney cancer, which typically cause substantial morbidity and mortality. Early detection and effective treatment are essential due to their high fatality rates. As a result, there is an urgent need for innovative research to improve the clinical management of patients with urologic cancers. A type of small noncoding RNAs of 22 nucleotides, microRNAs (miRNAs) are well-known for their important roles in a variety of developmental processes. Among these, microRNA-21 (miR-21) stands out as a commonly studied miRNA with implications in tumorigenesis and cancer development, particularly in urological tumors. Recent research has shed light on the dysregulation of miR-21 in urological tumors, offering insights into its potential as a prognostic, diagnostic, and therapeutic tool. This review delves into the pathogenesis of miR-21 in prostate, bladder, and renal cancers, its utility as a cancer biomarker, and the therapeutic possibilities of targeting miR-21.

## Introduction

Cancer of the urinary system, including prostate, bladder, and kidney cancer, is a significant contributor to morbidity and mortality globally. Prostate cancer, bladder cancer, and kidney cancer are among the most prevalent cancers of the urinary system, with prostate cancer being one of the top ten causes of cancer-related deaths in men in Europe. The latest global cancer statistics from 2020 report 1,414,259 new cases of prostate cancer, accounting for 7.3% of the total new cases, and 375,304 new deaths, representing 3.8% of the total new cases. Additionally, there were 573,278 new cases of bladder cancer (3.0%) and 212,536 new deaths (2.1%), as well as 431,288 new cases of kidney cancer (2.2%) and 179,368 new deaths (1.8%) (Sung et al., 2021). Despite advancements in prognostic tools for urologic tumors, long-term outcomes remain unfavorable due to issues such as drug resistance and recurrence. Addressing these challenges necessitates further research to identify novel biomarkers for precise monitoring of urologic tumor progression and to discover therapeutic targets that can enhance survival rates. Short, single-stranded RNAs, known as microRNAs (miRNAs), are naturally occurring molecules that interact with target messenger RNAs (mRNAs) to regulate gene expression at the post-transcriptional level (Lujambio and Lowe, 2012). Research has linked abnormalities in miRNAs to various diseases, particularly cancer. Oncogenic miRNAs (onco-miRs) can drive tumor progression, while anti-cancer miRNAs may impede cancer growth and metastasis by inhibiting oncogenes (Rupaimoole and Slack, 2017; Wang X. et al., 2021). These miRNAs can influence critical cellular processes such as cell proliferation, differentiation, and apoptosis (Maatouk and Harfe, 2006; Bueno et al., 2008; Wang and Lee, 2009). Among the numerous miRNAs associated with cancer development, miR-21 was one of the first identified as an oncogenic miRNA or “oncomiR.” Changes in miR-21 expression have been linked to disruptions in epigenetic factors, as well as transcriptional and post-transcriptional regulators, either during their biogenesis or through repression, ultimately leading to an oncogenic phenotype (Fujita et al., 2008; Feng and Tsao, 2016). This review aims to explore the regulatory role and function of miRNA-21 in prostate, renal cell, and bladder cancers, focusing on highly dysregulated signaling pathways. Recent advances in miRNA-21-based cancer therapies are summarized, highlighting the significant progress made since the discovery of miRNA-21’s critical regulatory function in various tumor types. Special attention is given to the newly identified role of miRNA-21 in carcinogenesis and its potential implications for the diagnosis and treatment of urological tumors, including prostate, kidney, and bladder cancers.

## MicroRNA

miRs are approximately 22-nucleotide long single-stranded noncoding RNAs. The discovery of the first miRNA, lin-4, in *Caenorhabditis elegans* in 1993 marked the beginning of understanding miRNA processing and function, leading to the identification of thousands of miRNAs across species (Lee et al., 1993; Wightman et al., 1993; Ambros, 2004). miRNA genes are distributed across the genome (Rodriguez et al., 2004), with many being non-coding genes that produce miRNA as their sole transcription product. In other instances, miRNAs are situated within introns or untranslated regions (UTRs) of protein-coding genes. Transcription of miRNA host genes by RNA polymerase II yields pri-miRNA or primary miRNA transcripts (Lee et al., 2004; Bortolin-Cavaillé et al., 2009). These pri-miRNAs typically undergo splicing, capping, and polyadenylation similar to protein-coding mRNAs (Cai et al., 2004). To become mature active miRNAs, pri-miRNAs undergo two nucleic acid endonuclease processing steps (Kim et al., 2009). The RNA-binding protein DGCR8 is associated with Drosha enzymes and is necessary for pri-miRNA cleavage (Han et al., 2004). The nucleic acid endonuclease Dicer cleaves the loop region of the precursor, releasing the mature miRNA (Hutvágner et al., 2001). Similar to Drosha, Dicer is linked to RNA-binding proteins. miRNAs ultimately become part of a RISC (or miRISC). The specific composition of this protein complex is currently unknown, but it includes the crucial protein Argonaute, with four family members identified in humans (Ago1-4) (Tabara et al., 1999; Hammond et al., 2001). Argonaute directly binds mature miRNAs and targets mRNAs that are complementary to the miRNA (Lewis et al., 2003). These molecules play crucial roles in biological processes by regulating gene expression at the post-transcriptional level. By binding to messenger RNAs in the cytoplasm, miRNAs can either degrade mRNA or temporarily inhibit translation (Fabian et al., 2010). The downregulation of specific miRNAs results in the upregulation of corresponding proteins, and vice versa. Conversely, upregulation of miRNAs leads to decreased expression of target proteins. Additionally, miRNAs induce translational repression by binding to the 3′and 5′untranslated regions (UTRs) as well as the coding region of mRNAs. They also contribute to gene transcription by binding within the promoter region of genes (Broughton et al., 2016). miRNAs are estimated to regulate approximately 60% of protein-coding genes, with an average of 200 targets per miRNA (Krek et al., 2005; Friedman et al., 2009). This indicates their significant role in regulating various physiological and pathological cellular processes. miRNAs are key regulators in cell fate determination, proliferation, and cell death. They play significant roles in various metabolic pathways, including cholesterol and fatty acid metabolism, as well as pancreatic islet function and glucose metabolism (Fernández-Hernando et al., 2013). Notably, 522 virally encoded miRNAs have been discovered, with a particular emphasis on the herpesvirus family (Griffiths-Jones et al., 2006). Beyond their metabolic and viral connections, miRNAs also participate in diverse cellular functions like immune responses (Calame, 2007; Gantier et al., 2007), insulin secretion (Poy et al., 2004), neurotransmitter synthesis (Greco and Rameshwar, 2007), circadian rhythms (Cheng et al., 2007), and viral replication (Jopling et al., 2005). The biogenesis and function of miRNAs are tightly controlled processes, and dysregulation in miRNA production, availability, and target regulation has been linked to a range of human diseases, including cancer (Croce, 2009).

## MiRNAs and cancer

The role of miRNAs in cancer has been extensively studied, revealing dysregulation in many cancer types and stages through various mechanisms (Croce, 2009). Chromosomal abnormalities are a known cause of miRNA dysregulation in cancer, with tumorigenesis often linked to chromosomal aberrations like deletions, amplifications, and translocations. Computational analysis has revealed that a significant number of miRNAs are located within cancer-associated genomic regions or fragile sites in both humans and mice (Calin et al., 2004; Sevignani et al., 2007). Furthermore, epigenetic factors can impact miRNA expression, with CpG island hypermethylation in promoter regions leading to heritable transcriptional silencing of tumor-suppressor genes in many cancers. This gene silencing through DNA methylation is intricately connected to histone modifications. Computational simulations have identified CpG islands proximal to numerous miRNAs. Transcription factors can stimulate the production of miRNAs by activating the transcription of pri-miRNAs (Lehmann et al., 2008). Notably, many oncogenes or tumor suppressors function as transcription factors, and various miRNA-transcription factor interactions have been observed in cancer, including with proteins like p53, c-Myc, and E2F (Dews et al., 2006; He et al., 2007; Sylvestre et al., 2007). Apart from the transcription rate of pri-miRNAs, the steady-state levels of mature miRNAs are influenced by the efficiency of processing their precursors and subsequent stability (Thomson et al., 2006). In cancer, miRNAs finely tune the expression of oncogenes and tumor suppressors in response to extracellular signals (Pagotto et al., 2022). Oncogenic miRNAs negatively suppress tumor suppressor genes, promoting tumor development, influencing cell differentiation and proliferation timing, cell cycle exit, and regulating oncogene expression, particularly the Ras gene (Takamizawa et al., 2004; He et al., 2005; Iorio et al., 2005; Li et al., 2012; Palma et al., 2014). Tumor suppressor miRNAs inhibit cancer by regulating oncogenes and genes controlling cell differentiation or apoptosis, targeting oncogenes involved in cell differentiation, cancer invasion, apoptosis, proliferation, and metastasis (Iorio et al., 2005; Zhang et al., 2007; Iqbal et al., 2019). The main aspects of miRNA-regulated cancer biology include the following: Cell cycle regulators often act as oncogenes or tumor suppressors. The most typical example is the cell cycle inhibitor p27(Kip1). p27(Kip1) is a tumor suppressor that is expressed at low levels in some cancers. p27(Kip1) binds to Cdk2-cell cycle protein E and blocks the transition from G1 to S. p27(Kip1) is a direct target of miR-221 and -222 in glioblastoma (Gillies and Lorimer, 2007; le Sage et al., 2007)and prostate cancer cells (Galardi et al., 2007); apoptosis is an active process controlled by gene expression programs. Apoptosis is an active process controlled by a gene expression program that varies depending on the biological context. miRNAs are involved in tumorigenesis by directly targeting anti-apoptotic genes. Representative examples are miR-29b (Mott et al., 2007) and miR-34s (Bommer et al., 2007), -15a and -16 (Cimmino et al., 2005) that inhibit the anti-apoptotic genes Mcl-1 and Bcl-2, respectively; malignant tumors, unlike benign tumors, are characterized by invasiveness and metastasis. Ectopic expression of miR-125 has been reported to impair cell motility and invasion in breast cancer cell lines (Scott et al., 2007); the recruitment of the vascular system is critical for tumor cell survival, and stimulation of neovascularization by c-Myc involves downregulation of the anti-angiogenic factor Tsp-1 (platelet reactive protein-1). c-Myc inhibits Tsp-1 and related proteins through activation of miR-17-92 cluster. CTGF (connective tissue growth factor). Tsp-1 and CTGF appear to be direct targets of miR-19 and -18 in this cluster, respectively. Ectopic expression of the miR-17-92 cluster is sufficient to promote angiogenesis (Dews et al., 2006). These properties have sparked significant interest in miRNAs as prognostic markers and therapeutic targets for human tumors (Croce, 2012).

## MiR-21 expression and urologic tumors

miR-21 stands out among the numerous miRNAs linked to cancer progression as one of the earliest identified oncogenic miRNAs. Positioned on chromosome 17q23.2 within the intron of the transmembrane protein 49 (TMEM49)/vesicular membrane protein 1 (VMP1) gene, miR-21 possesses a distinct and highly conserved promoter region. This region is known to be activated by activator protein 1 (AP-1), which interacts with the switch/sucrose nonfermentable (SWI/SNF) complex, as well as the Ets-associated proteins PU.1 and CCAAT/enhancer-binding protein (C/EBP). Additionally, miR-21 is influenced by nuclear factor I (NFI), serum response factor (SRF), p53, and signal transducer and activator of transcription 3 (STAT3) (Fujita et al., 2008; Feng and Tsao, 2016). miR-21 has been identified as significantly overexpressed in various human cancer types, such as breast, gastric, lung, esophageal, colorectal, biliary tract, nasopharyngeal, hepatocellular carcinomas, osteosarcomas, gliomas, leukemias, retinoblastomas, and lymphomas (Volinia et al., 2006; Wu et al., 2015). Among them is growing evidence that miR-21 functions as an oncogene. Recent literature increasingly highlights the association between urological tumors and miR-21, with numerous studies emphasizing its impact on prostate, bladder, and kidney cancers - three of the most prevalent urological malignancies. In the context of prostate cancer and miR-21 research, Guan et al. discovered that levels of miR-21 were notably higher in prostate cancer (PCa) tissues compared to adjacent noncancerous prostate tissues (Guan et al., 2016); Meanwhile, Kamla et al. illustrated that miR-21 plays a role in promoting prostate cancer stem cells (PCSC) and potentially targets apoptotic genes involved in the development of PCa, suggesting its potential as a diagnostic biomarker for the disease (Shukla et al., 2023); Furthermore, existing literature acknowledges the close association between the androgen receptor and hormone-dependent prostate cancer. Studies have also indicated that miR-21, recognized as an androgen receptor (AR)-regulated miRNA, can enhance the growth of androgen-dependent CaP and contribute to resistance to desmoplasia (Ribas et al., 2009). In the literature on bladder cancer and miR-21, Zhang et al. (2015) demonstrated that miR-21 mRNA expression was significantly reduced in bladder cancer (BC) tissues compared to normal bladder tissues. They also suggested that the upregulation of miR-21 in BC may promote tumor progression (Zhang et al., 2015). Regarding renal cell carcinoma (RCC) and miR-21, Lv et al. (2013) found that miR-21 was significantly overexpressed in RCC tissues compared to adjacent normal tissues. They observed that miR-21 inhibitors inhibited cell growth by inducing apoptosis and cell cycle arrest in S phase (Lv et al., 2013); The metastasis of clear cell renal cell carcinoma (ccRCC) may be triggered by epithelial mesenchymal transition and mesenchymal stem cells, which also contribute to the development of primary tumors. Furthermore, miR-21 overexpression was linked to the formation of ccRCC spheroids, while reducing its expression could directly inhibit the proliferation of ccRCC cells (Cao J. et al., 2016); Studies have shown that the expression of miR-21 is associated with the survival of renal cancer patients, with the expression level correlating with the 5-year survival rate and disease stage. Notably, patients with low miR-21 expression had a 100% 5-year survival rate post-surgery, while only 50% of patients with high miR-21 expression survived (Zaman et al., 2012). These findings underscore the importance of further investigating the specific mechanisms of miR-21 in urological tumors and elucidating its role in the development and progression of these malignancies.

## Mechanism of MiR-21 associated with urologic cancers

### Mechanisms of action associated with miR-21 in prostate cancer

**FIGURE 1:**
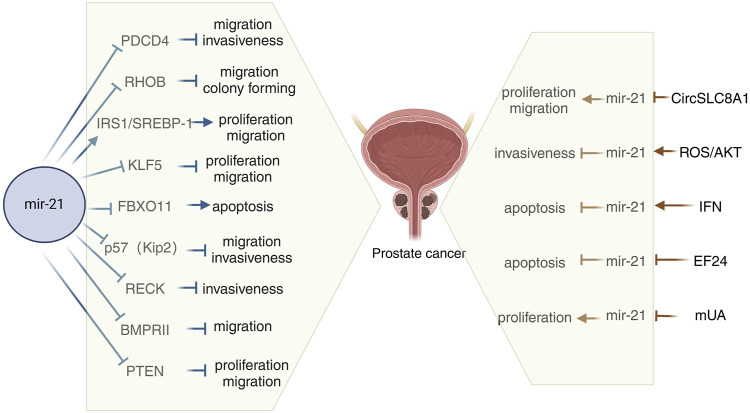
Schematic diagram of the mechanism of miR-21 in prostate cancer. mUA, Methylated urolithin A; PDCD4, Programmed cell death 4; SREBP-1, Sterol regulatory element binding protein 1; BMPRII, Bone morphogenetic protein receptor II.

In miR-21-related tumor-related studies, we have found that miR-21 can affect tumor progression both by regulating its downstream targets and by regulating its expression in tumors through related genes, thereby affecting tumor progression. On the right side of Figure 1, we summarize all current studies on the ability to influence prostate cancer progression by regulating miR-21 expression. Wang D. et al. (2021) conducted a study to assess the expression of CircSLC8A1 in human prostate cancer using qRT-PCR. Their findings indicated a significant reduction in CircSLC8A1 levels in PCa. The study also revealed that CircSLC8A1 acts as a tumor suppressor by impeding the proliferation and migration of PCa cells. Moreover, the upregulation of miR-21 in PCa suggests a potential direct interaction between miR-21 and circSLC8A1, with circSLC8A1 playing a role in inhibiting prostate cancer progression by sponging miR-21 (Wang D. et al., 2021). Jajoo et al. discovered that miR-21 plays a role in regulating the invasiveness of PC-3M-MM2 prostate cancer cells. They also found that the expression of miR-21 in these cells is influenced by high levels of reactive oxygen species (ROS) production. Previous research has demonstrated that ROS can impact invasiveness by activating Akt in prostate cancer cells, which in turn can regulate miR-21 expression. This suggests that Akt may serve as a target of ROS to modulate miR-21 levels (Kumar et al., 2008). Furthermore, Akt has been shown to directly influence miR-21 expression in prostate cancer cells (Sheth et al., 2012), indicating its potential role as a target for ROS-mediated regulation of miR-21. Overall, these findings suggest that ROS can stimulate the Akt pathway to enhance miR-21 expression (Jajoo et al., 2013). Interferons (IFN) are antiviral cytokines with profound impacts on cell functions such as proliferation, differentiation, apoptosis, and immune responses. Research on prostate cancer cells has revealed that miR-21 acts as a critical factor in suppressing IFN-induced apoptosis. Studies have indicated that the oncogenic miR-21 can be increased by IFN and that modulating miR-21 expression, particularly through miR-21 knockdown, can amplify the apoptotic effects of IFN (Yang et al., 2010). EF24, a curcumin analog, exhibits superior anticancer activity compared to curcumin. Research indicates that EF24 triggers apoptosis in ovarian, gastrointestinal, and breast cancer models (Adams et al., 2005; Liang et al., 2011; Zhu et al., 2012). Some studies have demonstrated EF24’s efficacy against prostate cancer *in vivo* by inhibiting miR-21 expression and upregulating miR-21 target genes for tumor suppression (Yang et al., 2013). Urolithin, a metabolite derived from ellagic acid-derived and produced by human colonic microflora, exhibits biological activity. Research investigating the impact of methylated urolithin A (mUA) on the viability of human prostate cancer DU145 cells has revealed a decrease in miR-21 expression following mUA exposure. Additionally, mUA has been found to suppress cell viability in DU145 cells by regulating miR-21 and its downstream targets such as PTEN, Akt, and Wnt/β-catenin signaling pathways (Zhou et al., 2016) (Figure 1).

On the left side of Figure 1, we summarize all the current studies on miR-21 affecting prostate cancer progression by modulating its downstream targets. Programmed cell death 4 (PDCD4) is a known suppressor of tumorigenesis, tumor progression, and invasion, functioning both independently and in response to external stimuli. PDCD4 is recognized as the first specific translational repressor to be characterized (Hilliard et al., 2006). Studies have shown that PDCD4 protein is predominantly localized in the cytoplasm, and that lower levels of PDCD4 expression are correlated with less differentiated prostate cancer. Additionally, miR-21 has been found to enhance the growth of prostate cancer cells by reducing the expression of PDCD4 (Dong et al., 2015). Tumor hypoxia is a well-recognized factor in the progression of prostate cancer and is known to influence the expression of various miRNAs. Research has shown that hypoxia plays a crucial role in the upregulation of miR-21 in prostate tumors, leading to the downregulation of the tumor suppressor gene Ras homologue family member B (RHOB) and ultimately promoting the progression of prostate cancer (Angel et al., 2023). Sterol regulatory element binding protein 1 (SREBP-1) plays a crucial role in adipogenesis and lipid metabolism, impacting disease progression and prognosis in prostate cancer (PCa) patients. Research indicates that SREBP-1 is regulated by miR-21 at a transcriptional level in cell cultures and mouse models, leading to increased cell proliferation, migration, and SREBP-1 levels in human PCa cells. The activation of the IRS1/SREBP-1 axis by miR-21 contributes to PCa advancement, suggesting that targeting the miR-21/SREBP-1 signaling pathway could be a promising approach to managing PCa aggressiveness (Kanagasabai et al., 2022). KLF5, a transcription factor located in the nucleus, plays a crucial role in regulating gene expression and impacting various cellular functions in cancer (Gao et al., 2015). Chen et al. discovered that miR-21, acting as an oncogene in PCa, targets KLF5 directly. Overexpression of miR-21 in LNCaP cells led to decreased levels of KLF5 mRNA and protein compared to MOCK or untreated controls. Conversely, reducing miR-21 in DU145 and PC-3 cells resulted in increased KLF5 mRNA and protein expression. The direct targeting of KLF5 by miR-21 promotes proliferation, migration, invasion, and resistance to apoptosis in both androgen-dependent and nondependent PCa cells (Guan et al., 2019). FBXO11, a component of the SKP1-CUL1-F-box ubiquitin ligase complex, is involved in targeting proteins for ubiquitination and proteasomal degradation. Through microarray analysis and quantitative PCR, one study identified and validated FBXO11 as a target gene of miR-21, an oncogenic miRNA. The study showed that miR-21 promotes tumorigenesis by inhibiting the expression of FBXO11, which typically functions as a tumor suppressor (Yang C. H. et al., 2015). The cell cycle protein-dependent kinase inhibitor p57(Kip2) is considered a potential oncogene linked to Beckwith-Wiedemann syndrome and sporadic cancers. Recent studies have identified p57(Kip2) as a target of miR-21 in prostate cancer, shedding light on a new oncogenic role of this miR-21. miR-21 has been found to reduce p57(Kip2) expression by targeting the gene’s coding region and dampen p57(Kip2)-mediated cellular functions in prostate cancer (Mishra et al., 2014). Research has demonstrated that miR-21 modulates cellular invasiveness by directly regulating the MMP inhibitor RECK, a protein rich in cysteine and featuring a Kazal motif, known for inhibiting multiple MMPs. Notably, the expression levels of RECK can predict the prognosis of various common cancers; lower RECK levels are often linked to increased invasiveness and a poorer prognosis (Takenaka et al., 2004; Kotzsch et al., 2005). Investigations on whether RECK is a target of miR-21 in the DU-145 prostate cancer cell line have revealed that miR-21 directly inhibits RECK and plays a significant role in the progression of prostate cancer by controlling RECK (Reis et al., 2012). Bone morphogenetic proteins (BMPs) belong to the tumor growth factor (TGF)-ß superfamily (Li, 2008). BMP acts by interacting with a diverse receptor complex composed of two types of serine-threonine kinase transmembrane receptors. BMPRII functions as a type 2 receptor for BMP, and mutations in the BMPRII gene are associated with the development of hereditary pulmonary arterial hypertension. Knocking down the BMPRII gene results in early embryonic death (Hassel et al., 2004; Rigelsky et al., 2008). Qin et al. discovered that bone morphogenetic protein receptor II (BMPRII) is directly regulated by miR-21, and they demonstrated a negative correlation between the protein levels of BMPRII and the abundance of miR-21 in PC3 and Lncap cells (Qin et al., 2009). The oncogene PTEN, located on chromosome 10, encodes a protein with lipid phosphatase and protein phosphatase activities. This protein dephosphorylates the PI3K 3-phosphorylation site in cells, inhibiting the phosphorylation of the downstream signal transduction molecule Akt. Research has shown that miR-21 can target and inhibit the expression of PTEN, enhancing the PI3K/Akt signaling pathway in prostate cancer cells. This ultimately promotes the proliferation and invasion of prostate cancer cells (Liu et al., 2011; Yang Y. et al., 2017). Taken together these findings add to the current understanding of the molecular processes involved in the development of prostate cancer and increase the likelihood of using miR-21 as a therapeutic target for prostate cancer.

### Mechanism of action associated with miR-21 in bladder cancer

**FIGURE 2:**
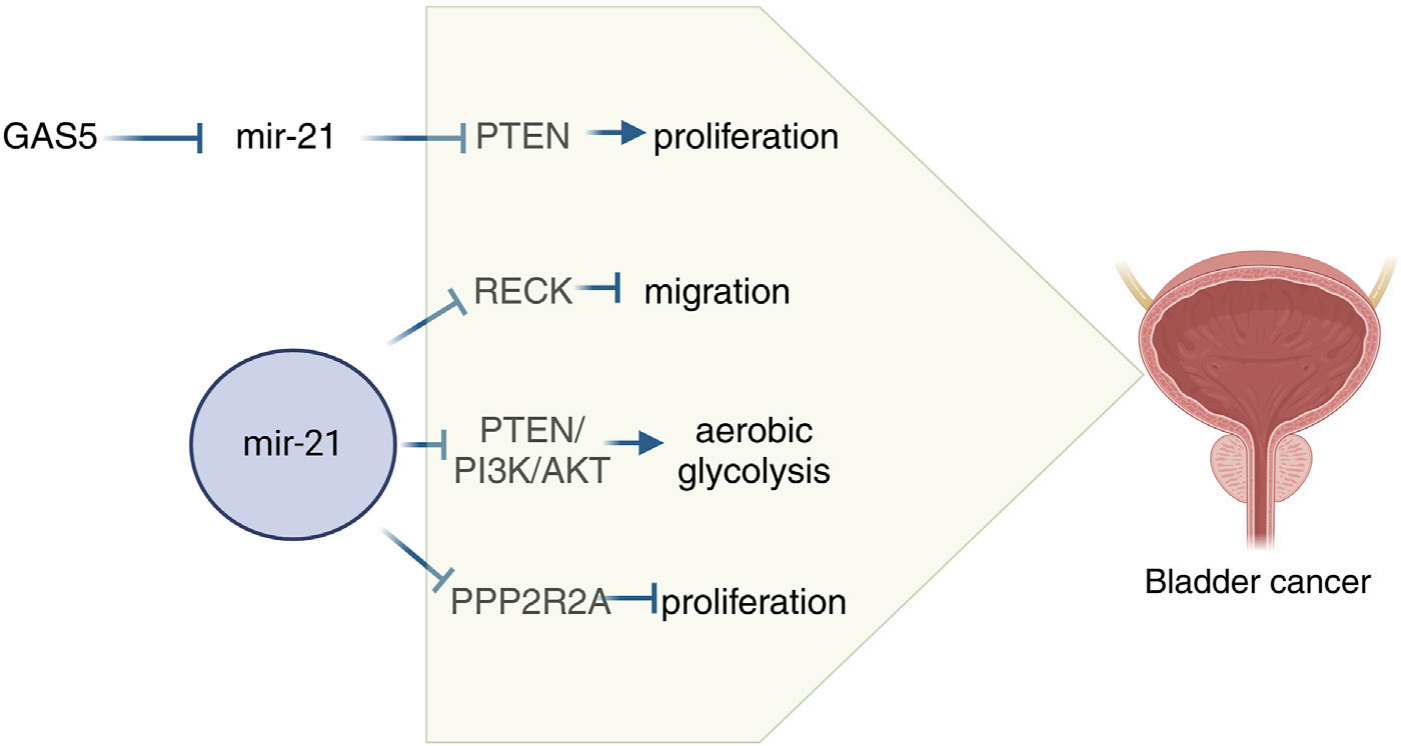
Schematic diagram of the mechanism of miR-21 in bladder cancer. GAS5, Growth arrest-specific transcripts 5; PPP2R2A, Protein phosphatase 2 regulatory subunit Balpha.

Growth arrest-specific transcript 5 (GAS5) is located at chromosome 1q25 and acts as a tumor suppressor (Pickard et al., 2013). Recent studies have revealed that GAS5 is downregulated in bladder cancer, leading to increased cell proliferation through CDK6 regulation (Liu Z. et al., 2013). Additionally, GAS5 has been found to inhibit metastasis of hepatocellular carcinoma cells by suppressing miR21 (Hu et al., 2016). A study investigating the interplay between GAS5 and miR-21 in bladder cancer cell reversal mechanisms showed that low GAS5 levels and high miR-21 levels were associated with bladder cancer. It was demonstrated that GAS5 directly targets miR-21 through luciferase analysis, while miR-21 targets PTEN. The study further revealed that low GAS5 expression upregulates miR-21, which in turn downregulates PTEN. Dual luciferase reporter gene assays confirmed PTEN as a direct target of miR-21. Overall, GAS5 exerts anti-proliferative and pro-apoptotic effects on bladder cancer cells by modulating the miR-21/PTEN pathway (Chen et al., 2020). RECK is a tumor suppressor gene that plays a role in inhibiting metalloproteinases, such as MMP9. Several studies have shown that inhibiting miR-21 expression through the transfection of a specific miR-21 inhibitor (anti-miR-21) leads to increased RECK expression, decreased MMP9 expression, and impacts the migration and proliferation of bladder cancer cells by modulating this pathway (Dos Santos et al., 2024). Macrophages are a prominent stromal cell type in the tumor microenvironment (TME), with the ability to exhibit either an immunological M1 phenotype that suppresses tumors or an M2 phenotype that promotes tumor inflammation and immunosuppression (Sica and Bronte, 2007). In a study conducted by Lin et al. (2020), it was found that miR-21 downregulated PTEN expression in macrophages, leading to the activation of the PI3K/AKT-mediated STAT3 signaling pathway, thereby promoting bladder cancer progression. A related study also highlighted the significance of the miRNA-21-mediated PTEN/PI3K/AKT pathway in bladder cancer (Yang X. et al., 2015). Marina et al. conducted a study that integrated computational and transcriptomic analyses in 28 bladder cancer cell lines. The study revealed a correlation between the protein phosphatase 2 regulatory subunit Balpha (PPP2R2A) and miR-21 levels. It was demonstrated that PPP2R2A is a direct target of miR-21 and plays a role in regulating the ERK pathway and the growth of bladder cancer cells. The findings suggest that miR-21 promotes tumor growth by inhibiting PPP2R2A expression and activating the ERK pathway (Koutsioumpa et al., 2018). Taken together these findings add to the current understanding of the molecular processes involved in the development of bladder cancer and increase the likelihood of using miR-21 as a therapeutic target for bladder cancer (Figure 2).

### Mechanism of action associated with miR-21 in kidney cancer

**FIGURE 3:**
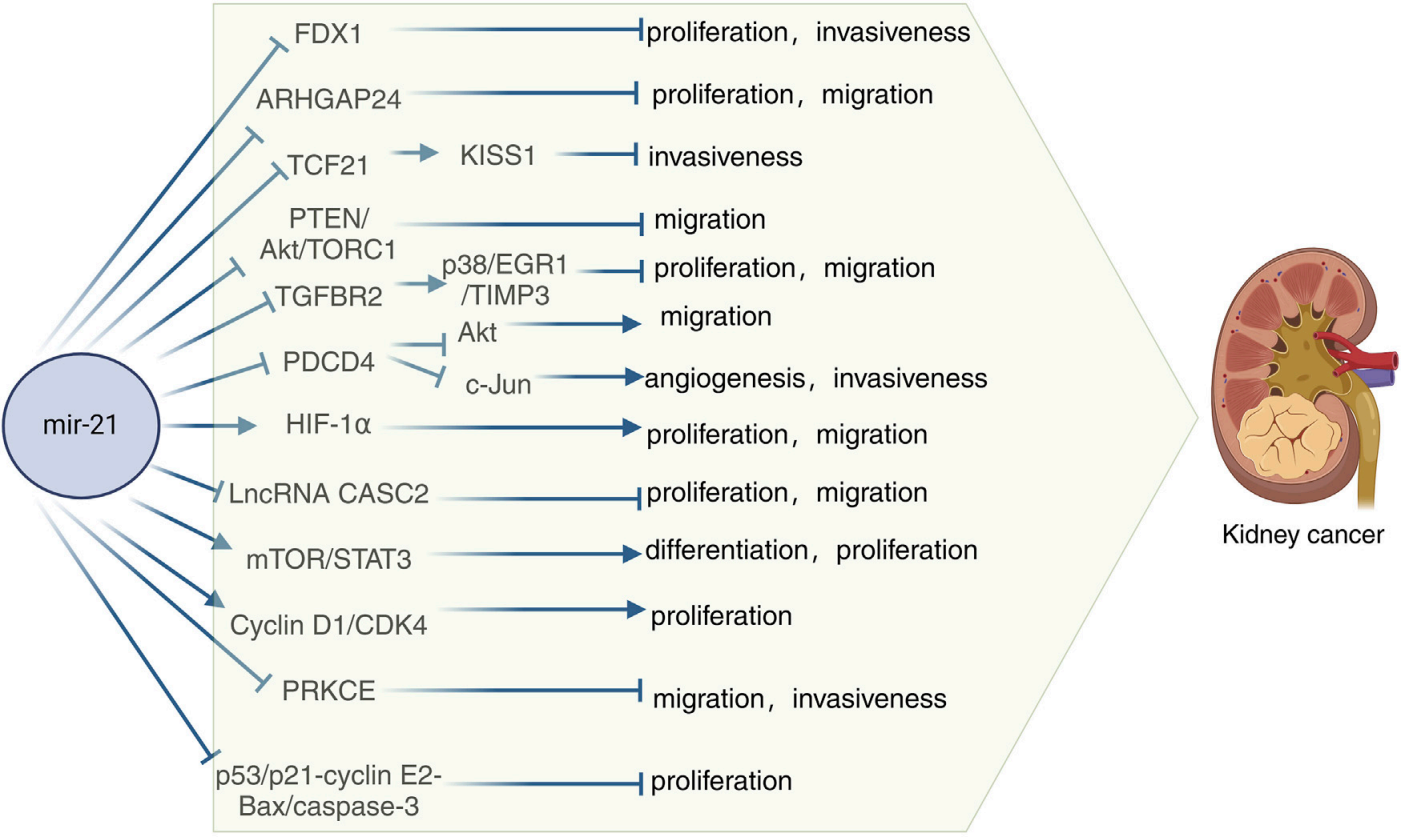
Schematic diagram of the mechanism of miR-21 in kidney cancer. TIMP3, Metalloproteinases 3; PTEN, Phosphatase and tensin homolog; PDCD4, Programmed cell death 4; HIF-1α, Hypoxia-inducible factor-1α; IncRNA CASC2, Long-stranded non-coding RNA Cancer susceptibility candidate gene 2; Cyclin D1, Cycle protein D1; PRKCE, Encodes protein kinase C ε.

FDX1, a key regulator of copper metabolism, was initially identified as an electron transfer protein that plays a role in the biosynthesis of steroid hormones, vitamin D, and bile acids in the urinary tissues, kidney, and liver, respectively (Sheftel et al., 2010). Recent research has highlighted the significance of FDX1 in copper-related tumorigenesis and its involvement in the progression of various cancers. A study demonstrated that FDX1 suppressed cell growth and invasion in ccRCC cells, with or without mammary gland formation. Furthermore, a luciferase activity assay indicated a negative correlation between miR-21-5p and FDX1, suggesting that miR-21-5p acts as an upstream regulator of FDX1 in driving ccRCC development (Xie et al., 2022). RhoGAP 24 (ARHGAP24) is a member of the RhoGAP family of proteins known for their oncogenic potential. Yang et al. discovered a close association between ARHGAP24 and hepatocellular carcinoma (Yang et al., 2022). In a study on RCC, Meng et al. observed elevated levels of miR-21-5p in RCC tissues, along with significantly reduced expression of ARHGAP24. They found that miR-21-5p, a known stimulator in RCC, exacerbated the cancer by suppressing the expression of its downstream target gene, ARHGAP24 (Meng et al., 2022). Using real-time fluorescence quantitative polymerase chain reaction, Zhang et al. discovered that miR-21 expression was elevated in human RCC specimens compared to normal renal cell specimens. Upregulation of Pre-miR-21 led to decreased expression of its direct target gene TCF21 and downregulation of KISS1 protein (Zhang et al., 2012). Tumor-associated macrophages (TAM) are classified as M2 and possess various pro-tumorigenic functions (Mohapatra et al., 2021; Munir et al., 2021). TAM are present in all solid tumors, including RCC, and are linked to RCC progression (Pusztai et al., 2019). Exosomes, which are small double-layered membrane vesicles ranging from 30–150 nm in diameter, play a crucial role in intercellular communication by transporting molecules such as mRNAs, miRNAs (miRNAs), long-chain non-coding RNAs, and proteins (Kalluri and LeBleu, 2020). For instance, exosomes derived from M2 macrophages (M2-Exos) were shown to boost the migration and invasion of colorectal cancer cells by transferring miR-21-5p and miR-155-5p (Lan et al., 2019). A study on M2-Exos and miR-21-5p revealed that M2-Exos facilitated the migration, invasion, and EMT of RCC cells, with miR-21-5p in M2-Exos promoting RCC metastasis by inhibiting PTEN/Akt signaling (Zhang et al., 2022). Another study demonstrated that increased miR-21 reduced PTEN protein levels, leading to Akt activation, which then inactivated tuberin to enhance TORC1 activity, resulting in the proliferation, migration, and invasion of renal cancer cells (Dey et al., 2012). Chen et al. discovered that miR-21 directly targets metalloproteinase 3 (TIMP3), which is overexpressed in RCC and acts as a tumor oncogene by promoting cancer cell proliferation and invasion (Chen et al., 2017). Another study revealed that miR-21 may impact the prognosis of hypertensive ccRCC patients. In the ccRCC microenvironment, tissue inhibitor of TIMP3, secreted mainly by tumor endothelial cells (TEC), is downregulated in tumor tissues of hypertensive ccRCC patients. Additionally, overexpression of TIMP3 has been shown to inhibit ccRCC proliferation and metastasis. The study also highlighted that hypertensive ccRCC patients exhibit reduced levels of circulating miR-21-5p, which leads to decreased TIMP3 expression in endothelial cells (EC) via the p38/EGR1 signaling axis (Wang et al., 2023). Programmed cell death 4 (PDCD4) is known to inhibit tumor transformation. Initially identified in a mouse epidermal cell system, the PDCD4 gene encodes a 64 kDa protein that is more abundant in tumor promoter-resistant cells compared to tumor promoter-sensitive cells undergoing transformation (Cmarik et al., 1999). Studies have linked the loss of PDCD4 expression to tumor progression in various cancers such as lung, colon, prostate, and breast (LaRonde-LeBlanc et al., 2007). Bioinformatics analyses have revealed that PDCD4 acts as a tumor suppressor by regulating processes like cell proliferation, invasion, metastasis, and tumor transformation through its interaction with miR-21 (LaRonde-LeBlanc et al., 2007; Selaru et al., 2009). In RCC, PDCD4 levels were found to be inversely related to miR-21 levels, with a normal renal cell line (HK-2) showing low miR-21 and high PDCD4 protein levels. The tumor suppressor function of PDCD4 is repressed by miR-21 at a post-transcriptional level, leading to increased proliferation, invasion, and metastasis in RCC (Li X. et al., 2014). In a related study, it was demonstrated that miR-21 downregulates PDCD4 at the post-transcriptional level, leading to enhanced cell colony formation and proliferation in a nude mouse model of renal cancer (Yuan et al., 2017). The interplay between PDCD4 and miR-21 in RCC has been further examined in subsequent research. Falguni et al. observed a notable rise in Akt phosphorylation in renal cancer cells, contributing to their proliferation and invasion, and investigated the regulatory role of miR-21 in Akt phosphorylation/activation (Dey et al., 2012). They also elucidated the involvement of PDCD4 in miR-21-mediated Akt phosphorylation, revealing that elevated miR-21 expression in renal cancer cells downregulates PDCD4 levels, resulting in Akt phosphorylation activation and facilitating metastatic adaptation (Bera et al., 2014). Additionally, Fan et al. reported that upregulation of miR-21 and downregulation of PDCD4 led to increased activator protein-1 (AP-1) phosphorylation in renal cancer cells. This alteration activated c-Jun within the AP-1 complex, further promoting the migration, invasion, and angiogenesis of renal cancer cells (Fan et al., 2020) (Figure 3).

Hypoxia-inducible factors-1α and 2-α (HIF-1α and HIF-2α) are transcription factors, with HIF-1α being notably abundant in the majority of ccRCC patients (Stoyanoff et al., 2016). Research has demonstrated that cresyl sulfate (pCS) triggers epithelial-mesenchymal transition (EMT), migration, and proliferation via the HIF-1α pathway. Moreover, pCS has been found to elevate miR-21 levels, promoting cell proliferation and EMT in ccRCC cells. Experimental findings indicate that blocking miR-21 through protein blotting techniques resulted in reduced HIF-1α expression in pCS-treated ccRCC cells (Wu et al., 2019). The long noncoding RNA (lncRNA) cancer susceptibility candidate gene 2 (CASC2), located on chromosome 10q26, was initially identified as downregulated in endometrial cancer, functioning as a tumor suppressor gene (Baldinu et al., 2004). In a study on CASC2 expression and function in RCC, it was discovered that CASC2 is a direct target of miR-21. MiR-21 was shown to decrease CASC2 expression in 786-O and A498 cells. Additionally, the overexpression of miR-21 partially reversed the inhibitory effects of CASC2 on cell proliferation and migration in 786-O and A498 cells (Cao Y. et al., 2016). Several studies have shown that the mTOR-STAT3 signaling pathway plays a role in the proliferation, differentiation, and apoptosis of miR-21 in human RCC cells (Liang et al., 2016). In a xenograft model, silencing miR-21 led to a significant inhibition of tumor growth and a decrease in STAT3 and hTERT expression, indicating that miR-21 regulates STAT3-mediated hTERT expression to modulate glioblastoma cell growth (Wang et al., 2012). This research highlights that miR-21 activates the mTOR-STAT3 signaling pathway to promote the survival and differentiation of human RCC cells while reducing apoptosis (Liang et al., 2016). G1 cell cycle proteins, including cyclin D1, regulate cell progression from G1 to S phase to initiate DNA synthesis. Cyclin D1 activates the cyclin-dependent kinase CDK4 for cell cycle progression. Studies have shown that miR-21 plays a role in the transcription of cyclin D1, and by inhibiting cyclin D1 mRNA expression, miR-21 Sponge effectively controls renal cancer cell proliferation through the activation of cyclin D1/CDK4 activity. Therefore, this research demonstrates that miR-21 regulates the proliferation of renal cancer cells by modulating the activity of cell cycle protein D1/CDK4 (Bera et al., 2013). Protein kinase C (PKC) is a serine/threonine kinase that regulates various cellular processes, such as proliferation, apoptosis, cell survival, and migration. The gene PRKCE encodes protein kinase C ε (PKC-ε), which plays a role in multiple physiological functions (Gorin and Pan, 2009). In a study by Wang et al., it was observed that reduced expression of PRKCE was linked to a negative prognosis in clear cell renal carcinoma. Through cell invasion assays, they demonstrated that overexpression of PRKCE could inhibit the invasive capabilities of KIRC cells. Furthermore, their analysis suggested that has-miR-21-5p might be a key regulatory miRNA for PRKCE in KIRC, affecting immune infiltration and prognosis by suppressing PRKCE expression (Wang et al., 2022). In a study conducted on RCC A-498 cells, it was discovered that the overexpression of miR-21 resulted in increased cell proliferation, inhibition of apoptosis, and reduced caspase-3 activity. Furthermore, the study revealed that miR-21 overexpression not only promoted cell proliferation and suppressed apoptosis and caspase-3 activity but also downregulated the expression of p53, CDKN1A p21, E2 cell cycle protein, and Bax protein in A-498 cells. Overall, the study demonstrated that the upregulation of miR-21 expression influenced RCC proliferation and apoptosis via the p53/p21-cyclin E2-Bax/caspase-3 signaling pathway (Liu et al., 2017). Taken together these findings add to the current understanding of the molecular processes involved in the development of kidney cancer and raise the possibility of using miR-21 as a therapeutic target for kidney cancer.

## Biological pathways, diagnostic and prognostic biomarker role of MiR-21 in urologic cancers

In our investigation of the relationship between miR-21 and urological tumors, we observed that miR-21 influences multiple biological pathways in these tumors. Furthermore, we identified the clinical importance of miR-21 as a valuable diagnostic and prognostic indicator for urological tumors. Our findings regarding these associations are summarized in Table 1.

**TABLE 1:** MiR-21 as a diagnostic, prognostic and predictive biomarker for urologic tumors.

Cancer	Expression	Experimental methods	Experimental models	Functions	Targets	Types of biomarkers	Refs.
Prostate cancer	Upward	RT-qPCR,WB Immunohistochemistry protein blot Transwell Testing Luciferase assay	SCID mouse PC3 cells, DU145 cells B16 Mouse melanoma cells, Lncap cells RWPE-1 cells	Apoptosis, proliferation, invasion, metastasis, angiogenesis, epithelial mesenchymal transition	RHOB, KLF5, PTEN	Diagnostic, prognostic markers, predictors of biological recurrence	Liu et al. (2011); Coppola et al. (2013); Leite et al. (2015); Yang et al. (2016); Yang et al. (2017a); Guan et al. (2019); Shukla et al. (2023)
Bladder cancer	Upward	RT-qPCR, WB protein blot flow cytometry immunofluorescence	naked mouse HTB-9 cells T24 cells	Proliferation, apoptosis, migration, invasion	RECK,PI3K/AKT	Prognostic molecular markers, molecular markers of relapse	Zhou et al. (2014); Zhang et al. (2015); Mitash et al. (2017); Lin et al. (2020); Dos Santos et al. (2024)
Kidney cancer	Upward	RT-qPCR,WB IHC, ChIP immunoblotting Immunohistochemistry Transwell Testing, MTT protein blot	Caki-1 cells 786-O cells, A498 cells ACHN cells	Proliferation, apoptosis, differentiation, migration, invasion, angiogenesis, transforming capacity, immune infiltration	PDCD4, mTOR-STAT3, LncRNA-CASC2, PRKCE	Diagnosis, prognostic markers, lymph node metastasis, distant metastasis markers	Faragalla et al. (2012); Li et al. (2014a); Cao et al. (2016b); Liang et al. (2016); Yu et al. (2018); Fan et al. (2020); Wang et al. (2022)

RT-qPCR, Real-time reverse transcription and quantitative PCR; IHC, Immunohistochemistry; WB, Western blot analysis; MTT, Methylthiazolyldiphenyl-tetrazolium bromide; ChIP, Chromatin immunoprecipitation.

## Potential role of MiR-21 in the treatment of urologic tumors

### Using miR-21 as a gene related to urologic cancer treatment

**FIGURE 4:**
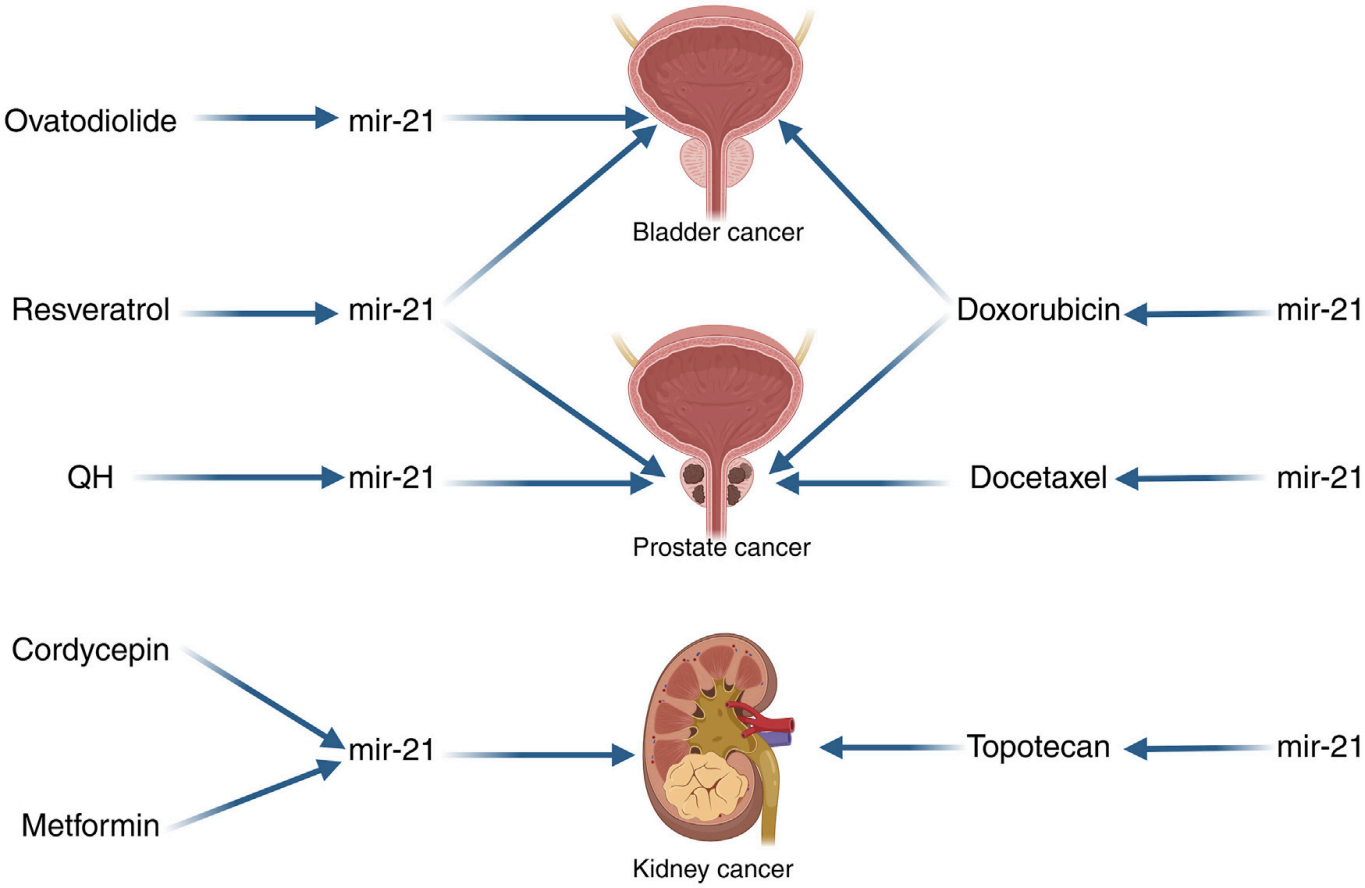
miR-21 Schematic diagrams related to the treatment of urologic cancers. QH, The combination of chrysin and quercetin; Note: The arrows in this image only indicate pointing action, not direct targeting. The left side of the image indicates that the drugs can affect the action of miR-21 in the tumor, and the right side of the image indicates that miR-21 can affect the sensitivity of the tumor to the drugs.

Tumor therapeutic resistance is a prevalent issue in cancer treatment, with the development of chemotherapeutic resistance posing a significant challenge. Despite advancements in cancer treatment, this remains a major obstacle. Research has indicated that miR-21 can impact the sensitivity of cancer cells to specific drugs. In our integration of the literature, we found that miR-21 was able to influence chemotherapy resistance in urologic tumors. Doxorubicin (DOX) is commonly used as a cytotoxic agent in the treatment of superficial and muscle-invasive bladder cancer, both intravesical and intravenous. This anthracycline antibiotic functions by intercalating into DNA, preventing resealing, halting replication, and ultimately leading to DNA destruction. In a study conducted by Tao et al., it was observed that upregulation of miR-21 resulted in decreased doxorubicin-induced apoptosis in T24 cells, while inhibition of miR-21 enhanced cell death. This suggests that miR-21 overexpression induces resistance to doxorubicin in T24 cell lines, whereas its downregulation sensitizes these cells to the drug (Tao et al., 2011). Furthermore, in a study examining the correlation between doxorubicin and prostate cancer, miR-21 was found to be highly expressed in PC3/DOX cells. Inhibition of miR-21 notably reduced the expression and activity of P-glycoprotein (P-gp) in DOX-resistant cells, thereby eliminating multidrug resistance (MDR) reversal by enhancing intracellular doxorubicin accumulation in PC3/DOX cells (Zhao et al., 2021). Prostate cancer is typically responsive to androgens at the time of initial diagnosis, leading to the administration of anti-androgen therapy for most patients. However, over time, patients may develop androgen-dependent prostate cancer (AIPC), for which docetaxel is the standard treatment (Pazdur et al., 1993). Research has shown that ectopic expression of miR-21 can increase resistance to docetaxel in PC3wt cells, while inhibiting miR-21 expression in PC3R cells can reduce resistance to docetaxel (Shi et al., 2010). Another study has indicated that miR-21 plays a role in the resistance of PC3 cells to docetaxel, suggesting that targeting miR-21 could be a promising therapeutic strategy to enhance the sensitivity of prostate cancer to docetaxel (Zhang et al., 2011). Naro et al. discovered an oxadiazole inhibitor of miR-21 through high-throughput screening. Further studies on structure-activity relationships revealed that the small molecule 37 is a strong inhibitor of miR-21 function. When miR-21 was inhibited in chemotherapy-resistant RCC cell lines using small molecule 37, the expression of tumor suppressor proteins was restored, leading to increased sensitivity to topotecan-induced apoptosis. This resulted in enhanced topotecan activity in cell viability and clone formation assays (Naro et al., 2018) (Figure 4).

Among the numerous treatments for cancer, none are able to completely eliminate cancer cells from a patient’s body, with the side effects of current treatment methods being a subject of concern. A more comprehensive comprehension of the molecular mechanisms of cancer has enabled us to target cells at their fundamental source. Given that miRNAs play a crucial role in regulating various key factors in tumorigenesis, the utilization of miRNAs for tumor therapy has garnered significant interest. This review outlines the ongoing research on therapies that impact urological tumors by modulating miR-21 and subsequently influencing them. M2 polarized tumor-associated macrophages (M2 TAM) have been implicated in the progression and drug resistance of bladder cancer (BCa). Wu et al. conducted experiments demonstrating that M2 TAM can induce malignant properties in BCa cells, partly through the release of oncogenic extracellular vesicles (EVs). Treatment with ovodiolactone (OV) was found to prevent the polarization of M2 TAM, reduce the EV cargo miR-21 derived from M2 TAM, and inhibit the β-catenin/mTOR/CDK6 signaling pathway. Overall, ovodiolactone was shown to inhibit bladder cancer progression by suppressing the mTOR/β-catenin/CDK6 pathway and exosomal miR-21 derived from M2 tumor-associated macrophages (Wu et al., 2020). Resveratrol, a dietary polyphenolic compound found in grapes and red wine, has been shown to inhibit the growth of bladder cancer cells and promote cell death. A recent study utilized real-time fluorescence quantitative PCR to demonstrate that resveratrol downregulated miR-21 expression and modulated the levels of Akt and Bcl-2 proteins, thereby influencing bladder cancer cell apoptosis through the miR-21-mediated Akt/Bcl-2 signaling pathway (Zhou et al., 2014). In another investigation focusing on resveratrol and prostate cancer, researchers observed a decrease in the expression of several prostate cancer-related miRNAs, including miR-21, in PC-3M-MM2 cells, an aggressive form of prostate cancer lacking androgen receptors. This finding suggested that resveratrol could impede the progression and spread of prostate cancer by targeting the Akt/miR-21 pathway. Overall, these studies highlight the potential of resveratrol in slowing down the advancement of prostate cancer through its effects on the Akt/miR-21 pathway (Sheth et al., 2012). The combination of chrysin and quercetin (QH; 1:1) has been shown to inhibit the growth of human leukemia cells (Mertens-Talcott et al., 2003). In a study conducted by Yang et al., QH was found to significantly reduce the invasion and migration of PC3 cells, as well as decrease the expression of various prostate tumor-associated miRNAs, including miR-21, when compared to untreated human prostate cancer cells. These results suggest that QH may act as an anticancer agent against human prostate cancer cells by targeting the miR-21 signaling pathway (Yang F. Q. et al., 2015). Cordycepin, an active ingredient derived from the traditional Chinese herb Cordyceps sinensis, has been found to exhibit antitumor activity across various cancer types. A study revealed that cordycepin downregulated extracellular signal-regulated kinase (ERK) and DUSP5, upregulated phosphorylated JNK (p-JNK), and triggered apoptosis. This research suggests that targeting ERK-JNK signaling with cordycepin-induced apoptosis could serve as a promising therapeutic approach for treating renal cancer (Hwang et al., 2016). Additionally, Zhao et al. demonstrated through quantitative real-time fluorescence quantitative PCR and protein blotting analysis that cordycepin reduced miR-21 expression and Akt phosphorylation levels in a dose-dependent manner, while increasing PTEN phosphatase levels in Caki-1 cells. Their findings indicate that cordycepin induces apoptosis in renal cancer cells by modulating miR-21 and PTEN phosphatase levels (Yang C. et al., 2017). Metformin (MF) is an antidiabetic drug that not only improves glycemic control but also affects various pathways in both normal and cancerous cells. This leads to the inhibition of cell proliferation, cell cycle arrest, and apoptosis (Stumvoll et al., 1995; Li W. et al., 2014; Hadad et al., 2014). Studies have shown that metformin can induce G0/G1 cell cycle arrest and hinder the growth of RCC both in laboratory settings and in living organisms (Liu J. et al., 2013). In a separate investigation on metformin’s impact on RCC, it was observed that MF treatment led to a decrease in miR-21 AMPK levels and an increase in PTEN expression in cell lines. The research highlighted that differences in the sensitivity of RCC cells to metformin were linked to the regulation of miR-21/PTEN expression, which subsequently influenced AKT signaling and ultimately affected the growth of RCC (Kalogirou et al., 2016).

## Discussion

MiRNAs are stably detected in plasma and serum, making them valuable molecular biomarkers for non-invasive cancer diagnosis and prognosis (Bartel, 2004; Cortez et al., 2011). MiRNAs can be found in biological fluids either within extracellular vesicles or as ribonucleoprotein complexes not associated with vesicles, and can help distinguish between various stages of disease progression (Creemers et al., 2012). Although methods for recognizing, utilizing, and inhibiting miR-21 have advanced in the past decade, there are still many unknown details, such as the factors influencing the formation of typical versus atypical isoomiR forms in specific cancers. Further exploration of these mechanisms and their implications will offer deeper insights into the role of miR-21 in cancer. It is now widely acknowledged that miRNAs operate in both intracellular and extracellular settings. Limited research has investigated the involvement of miR-21 in intercellular communication within the tumor microenvironment, particularly in patient or patient-derived tumor models. Given miR-21’s capability to target multiple tumor suppressor and oncogenic pathways, such investigations could uncover additional roles of miR-21 in disease progression. Various miRNA-targeted RNA therapies, including miravirsen, mesoMiR-1, and lademirsen, are currently undergoing clinical trials (Winkle et al., 2021). Different methods of miR-21 inhibition target specific steps in miRNA biogenesis. For instance, small molecule inhibitors like diazobenzene and estradiol have been utilized to target transcription in miRNA biogenesis (Gumireddy et al., 2008; Wickramasinghe et al., 2009; Fu et al., 2021). Additionally, direct targeting of miRNA structures using small molecules that bind to the G-hairpin of the hTERT-G-quadruplex-forming sequence has shown to downregulate expression and exhibit a strong anticancer effect in mice. Despite challenges, RNA therapy holds promise for clinical applications (Song et al., 2019). While advancements have been made in predicting interactions and developing therapeutic strategies to inhibit miR-21, challenges related to cancer heterogeneity and the complex microenvironment network have become more evident. It is now understood that therapy should take into account the intricate miRNA-mRNA, cellular protein regulator, and ncRNA networks to overcome current limitations in cancer treatment. With the growing knowledge of miR-21 and its role in cancer, there is hope for the development of safe and effective RNA-based therapies for clinical use.

## Summary and prospect

Recent advances in miRNA research on urologic tumors have revealed significant dysregulation of miRNAs in these cancer types, with implications for key carcinogenic pathways. Interestingly, we found that miR-21 is playing the role of an oncogene in prostate, bladder and kidney cancers in all the literature we have studied so far. This review focuses on the latest insights into the targeting of signaling pathways by miR-21. While the body of literature on miR-21 in urological tumors is expanding, a comprehensive overview of signal transduction regulation in these tumors is lacking. Therefore, this review consolidates existing findings on the role of miR-21 in regulating signaling pathways in urological tumors. The collective evidence suggests that miR-21 predominantly functions as a tumor suppressor, inhibiting cell proliferation, invasion, metastasis, and tumor growth across various urological cancer types. Many studies have shown that miR-21 is dysregulated in urologic tumors and is believed to target important pathways involved in carcinogenesis. There are various target genes and pathways linked to the function of miR-21, and although therapeutic strategies targeting miR-21 are still in early stages, progress has been made in recent years. This review aims to summarize the current findings on the therapeutic potential of miR-21 in urological cancers. Additionally, the potential interactions of miRNAs with other noncoding RNAs are being explored. Further research is needed to investigate the accuracy and specificity of miR-21 as a diagnostic biomarker, as well as the potential unintended effects of using anti-miR-21 as a therapeutic intervention. A more comprehensive understanding of miR-21, its target genes, and its molecular mechanisms of action will be crucial for successfully translating current research findings into clinical practice.
